# Does Despotic Leadership Harm Employee Family Life: Exploring the Effects of Emotional Exhaustion and Anxiety

**DOI:** 10.3389/fpsyg.2018.00601

**Published:** 2018-05-03

**Authors:** Shazia Nauman, Tasneem Fatima, Inam Ul Haq

**Affiliations:** ^1^Riphah School of Business & Management, Riphah International University, Lahore, Pakistan; ^2^Faculty of Management Sciences, International Islamic University, Islamabad, Pakistan; ^3^Lahore Business School, University of Lahore, Lahore, Pakistan

**Keywords:** despotic leadership, emotional exhaustion, anxiety, work-family conflict, life satisfaction

## Abstract

Research has not focused on the negative effects of despotic leadership on subordinates’ life satisfaction and the interface between work and family. Drawing on the Conservation of Resources theory, this research investigates the mediating effect of emotional exhaustion through which despotic leadership transcends from the workplace to subordinates’ personal lives, resulting in work-family conflict and decreased life satisfaction. The research also examines the moderating effect of subordinates’ anxiety on the relationship of their perceptions of despotic leadership with work-family conflict and life satisfaction. Three waves of time-lagged data was collected from 224 book sellers who work in publishing houses. We used Hayes’ PROCESS to test moderation and SEM to test mediation. The results of the study suggest that despotic leadership is related to work-family conflict via emotional exhaustion, but offer no support for its relationship with life satisfaction. As expected, when subordinates’ anxiety increases, the positive relationship between a supervisor’s despotism and his or her subordinates’ work-family conflict and the negative relationship between despotic leadership and life satisfaction both strengthen. The results suggest that despotic leaders harm their subordinates’ non-work lives, and these effects intensify when subordinates have high levels of anxiety. These findings have important implications for service organizations in mitigating the negative effects of despotic leadership by minimizing subordinates’ anxiety through coping mechanisms and giving reward and incentives.

## Introduction

Research has highlighted the negative or dark side of leadership (Griffin and Lopez, 2005; Wu and Hu, 2009; Naseer et al., 2016) by revealing destructive aspects of leadership that can have negative effects (Schyns and Hansbrough, 2010) on such factors as absenteeism, turnover, effectiveness (Tepper et al., 2006), emotional exhaustion (Harvey et al., 2007), deviant work behavior (Duffy et al., 2002), job satisfaction (Tepper, 2000; Tepper et al., 2004), stress (Tepper, 2000; Chen et al., 2009), and performance (Aryee et al., 2007). These destructive leadership behaviors have been conceptualized and examined under such labels as petty tyranny (Ashforth, 1994), abusive supervision (Tepper, 2000), destructive leadership (Schyns and Hansbrough, 2010), and despotic leadership (Aronson, 2001). According to Schyns and Schilling (2013), despotic leadership comprises prominent characteristics of negative leadership types, but there is a lack of research in this area in the management and psychology literatures (Naseer et al., 2016).

De Hoogh and Den Hartog (2008) defined despotic leadership as a leader’s tendency to engage in authoritarian and dominant behavior in pursuit of self-interest, self-aggrandizement, and exploitation of their subordinates. According to Schilling (2009), despotic leaders want unquestioned submission from their subordinates and use demanding and controlling mechanisms to manipulate and exploit their subordinates for personal gain, regardless of their subordinates’ needs and concerns. Thus, despotic leaders work against their organizations’ legitimate interests by indulging in self-serving and morally corrupt behavior (Aronson, 2001). Despotic leaders’ unethical and unfair behavior in the workplace negatively impacts subordinates’ job performance, organizational citizenship behaviors, and creativity (Naseer et al., 2016). Despite increasing evidence that despotic leadership is harmful to employees, there is a lack of research on its negative effects on employees’ life satisfaction and the interface between their work and family lives.

The effects of destructive leadership behavior may not be limited to subordinates, as they may also enfold the organization, customers, employees’ families, and even society in general. Research has indicated that such behavior is related to a number of negative outcomes, including lowered job satisfaction, organizational commitment, organizational performance, and increased emotional exhaustion, turnover intentions, work-family conflict, and psychological distress among employees (Richman et al., 1992; Ashforth, 1997; Tepper, 2000; Aasland et al., 2010; Hershcovis and Rafferty, 2012; Schyns and Schilling, 2013). Therefore, the negative aspects of leadership are a matter of grave concern for organizations (Hoobler and Hu, 2013) and further investigation is needed (Schyns and Schilling, 2013; Collins and Jackson, 2015) into what causes destructive leadership and how its negative aspects affect subordinates’ behavior and relationships at home. The current study addresses this research gap by investigating the harmful effects of despotic leadership on subordinates’ life satisfaction and work-family conflicts.

There are several reasons for focusing on these particular outcome variables. First, as despotic leadership is a social stressor and have harmful effects on the home life of a subordinate, we therefore choose work- family conflict instead of work life balance which is a more positive way of viewing work-family relationships. For example, according to Clark, work–family balance is “satisfaction and good functioning at work and at home with a minimum of role conflict” (Clark, 2000). In a similar vein, Frone (2003) refers work life balance as an absence of role conflict and presence of facilitation: “low levels of inter-role conflict and high levels of inter-role facilitation represent work–family balance.” Greenhaus et al. (2003) found that when individuals invest little time or involvement in their combined work and family roles, balance has little or no implications for an individual’s quality of life. Second, despotic leadership is a social stressor which is an antecedent of work family conflict and life satisfaction. Third, despotic leadership creates stressors like emotional exhaustion, which has been linked to employee well-being and quality of life at home (Ernst Kossek and Ozeki, 1998). Fourth, studies that have observed spillover effects from work to home show that job experiences influence the home life of employees even after they leave the workplace (Ilies et al., 2009; Eby et al., 2010; Wagner et al., 2014), which shows that the effects of emotional exhaustion may affect other domains of employees’ lives as well. For instance, emotional exhaustion harms the family domain, increases work- family conflict, and decreases life satisfaction (Gali Cinamon and Rich, 2010; Lambert et al., 2010; Carlson et al., 2012; Boekhorst et al., 2017). Here, we argue that these dependent variables are the most suitable for this study, as they directly influence the subordinates’ wellbeing and quality of life.

The Conservation of Resources (COR) theory comprises several stress theories (Hobfoll, 1989) and provides insight into the interface between work and family (Witt and Carlson, 2006; Grandey and Cropanzano, 1999). The COR theory suggests that people experience stress from an actual or threatened loss of resources (Hobfoll, 1989). It also envisages that resources are lost as individuals try to manage both work and family roles (Grandey and Cropanzano, 1999). This potential or real loss leads to conflicts in the interface between work and family (Witt and Carlson, 2006). According to Grandey and Cropanzano (1999), negative work stressors hamper subordinates’ ability to perform their family roles, which may result in inter-role conflict in the form of work-family conflict and life dissatisfaction.

Using the COR theory as a foundation, we theorized that despotic leadership is the source of social stress and the loss of leadership support reflected in self-serving behavior in the supervisor-subordinate relationship. As despotic leadership is authoritarian, vengeful, unethical, self-serving, and exploitative (De Hoogh and Den Hartog, 2008; Naseer et al., 2016), despotic leadership in a highly collectivist, uncertainty avoidant, and power distant culture like Pakistan would lead to emotional exhaustion (Hofstede, 1983, 2010). The loss of resources that results from emotional exhaustion also leads subordinates to experience decreased levels of life satisfaction and has a negative effect on the work-family interface. In this context, we posit that despotic leadership is directly related to the outcome variables of life satisfaction and work-family conflict and is indirectly related via emotional exhaustion.

According to Spielberger and Sydeman (1994), anxiety is defined as “the tendency to perceive a wide range of situations as dangerous or threatening” and is also specified as a predictor of victimization (Olweus, 1978; Aquino and Thau, 2009). As subordinates differ in their tendency to perceive authoritarian behavior in their leaders, their reactions vary such that the subordinates who have a high degree of anxiety are likely to be more sensitive than those who do not. Drawing from COR theory, subordinates lose the support of a despotic leader, and the leader’s self-serving behavior is likely to decrease subordinates’ life satisfaction and affect their work-family interface negatively. This loss intensifies among subordinates who have high levels of anxiety. Tepper (2007) also proposes that future research identifies personality moderators of destructive leadership. Since the perceivers’ personality affects their reactions to despotic leadership, and trait anxiety moderates the relationship between such leadership, life satisfaction, and work-family conflict, we contend that the interactive relationship between perceived despotic leadership and subordinates’ anxiety has a detrimental effect on the subordinates’ life satisfaction and work-family conflict.

Therefore, the purpose of this research is to examine the extent to which despotic leadership transcends the work boundary to affect employees’ life satisfaction and the interface between their work and family lives. Consequently, the current research investigates how despotic leadership creates emotional exhaustion, which influences employees’ life satisfaction and work-family conflict. The moderating role of anxiety on the relationship between despotic leadership and the outcome variables has been probed as well. Since the dark side of leadership is more obvious in a highly collectivist and power distant culture (Luthans et al., 1998) as subordinates in high power-distant and collectivistic cultures are expected to obey what their supervisors order without questioning and accept power inequalities, we thus see Pakistani employees as ideal for this study (Naseer et al., 2016).

## Literature Review and Hypotheses

### Despotic Leadership and Emotional Exhaustion

Emotional exhaustion occurs when emotional demands exceed an individual’s ability to deal with interpersonal interactions at work (Maslach et al., 2001). There is growing evidence that aggressive leadership leads to harmful outcomes for subordinates, including anxiety, depression (Tepper, 2000), and burnout (Tepper, 2000; Harvey et al., 2007; Aryee et al., 2008; Wu and Hu, 2009). Despotic leaders, who are autocratic, inconsiderate, and exploitative, create stress among their subordinates, resulting in burnout (Ashforth and Lee, 1997; Den Hartog and De Hoogh, 2009; Schilling, 2009; Fontaine et al., 2010). Therefore, we hypothesize:

H1: Subordinates’ perceptions of despotic leadership are positively related to their emotional exhaustion.

### Despotic Leadership and Work-Family Conflict

Despotic leaders are unethical and authoritarian, use an unethical code of conduct, and have little regard for others’ interests (Naseer et al., 2016). In pursuing their self-interests, they can be domineering, controlling, vengeful, and exploitative (Bass, 1990; Howell and Avolio, 1992; Aronson, 2001). The harmful consequences of despotic leadership highlight the importance of understanding the effects of this kind of leadership on subordinates’ lives. Work-family conflict has been defined as “a form of inter-role conflict in which the role pressures from the work and family domains are mutually incompatible in some respect” (Greenhaus and Beutell, 1985, p. 77), suggesting that “participation in the work (family) role is made more difficult by virtue of participation in the family (work) role” (Van Steenbergen et al., 2009; Kalliath et al., 2012). We examine work-family conflict since the emphasis of the study is on assessing despotic leadership as a social stressor that encircles the work and family life of the subordinates.

Work family conflict has been conceptualized into three types: time-based, strain-based, and behavior-based. Time-based conflict arises when the time committed to one role makes it difficult to participate in another role, such as when one has no time to participate in family functions or children’s school events because of time spent at work. An example of strain-based conflict is being too tired from work to do home chores or help one’s spouse. Behavior-based conflict occurs when one’s emotional exhaustion from work leads to coming home in a bad mood and fighting with one’s spouse.

According to Hoobler and Brass (2006), subordinates carry workplace aggression home in the form of behaviors that undermine their families. In a similar vein, Demsky et al. (2014) found a positive relationship between workplace aggression and work- family conflict. According to (Westman, 2001), despotic leadership increases tension in subordinates’ marital relationships, weakening the family structure (Carlson et al., 2011). Despotic leaders demand unquestioned compliance and obedience from their subordinates (Schilling, 2009), are self-centered, have low ethical standards (De Hoogh and Den Hartog, 2008), and exploit their subordinates for personal gain (Naseer et al., 2016). Therefore, we argue that despotic leadership is a workplace stressor that leaves the subordinates drained and emotionally exhausted, dramatically stressing their personal lives. Therefore, we hypothesize that:

H2a: Subordinates’ perceptions of despotic leadership are directly and positively related to their work-family conflict.

### Despotic Leadership and Life Satisfaction

Life satisfaction is a critical indicator of an individual’s overall well-being from evaluating his or her life (Karatepe and Baddar, 2006; Aryee et al., 2007; Erdogan et al., 2012); life satisfaction is widely accepted as a vital factor in an individual’s quality of life (Pavot and Diener, 1993). There is ample evidence to support the strong relationship between experience at work and an individual’s overall perspective of his or her life (Rain et al., 1991). Considering the significance of life satisfaction in measuring an individual’s wellbeing, a few studies have examined the harmful effects of negative leadership on life satisfaction and have suggested that poor and unfair treatment by others in the form of abusive supervision (Tepper, 2000) and workplace bullying (Dzuka and Dalbert, 2007; Moore et al., 2012) is negatively related to life satisfaction. Following this line of discussion, we posit that, when a leader is manipulative, exploitive, and vengeful, subordinates’ sense of personal control to cope up with such pressures declines. As a result, subordinates feel emotionally exhausted and are likely to have low level of life satisfaction. Therefore, we hypothesize that:

H2b: Subordinates’ perception of despotic leadership is directly and negatively related to their life satisfaction.

### Emotional Exhaustion as a Mediator

According to Westman et al. (2004), burnout is a significant predictor of work-family conflict. Emotional exhaustion, one of the core factors in burnout (Johnson and Spector, 2007), is most clearly linked to depletion of resources, as described by COR theory. Drawing from COR theory, we posit that despotic leadership may lead subordinates to deplete their personal and emotional resources and become exhausted, an effect that is likely to increase over time as the frequency of interaction with the supervisor increases (Grandey et al., 2004). Emotionally exhausted subordinates may have little energy left for family chores or family enriching activities, leading to work-family conflict (Gali Cinamon and Rich, 2010; Carlson et al., 2012). Using COR theory as a foundation, we observed that subordinates’ experience of despotic leadership results in depletion of subordinates’ energy, increasing emotional exhaustion and work-to-family conflict and diminishing life satisfaction. Therefore, we hypothesize:

H3: Subordinates’ emotional exhaustion is (a) positively related to work-family conflict and (b) negatively related to life satisfaction.

H4: Subordinates’ perceptions of despotic leadership are (a) positively related to work family conflict and (b) negatively related to life satisfaction via emotional exhaustion.

### Anxiety as a Moderator

Personality has been identified as an antecedent of work-family conflict (Michel et al., 2011), and work-family researchers have called for an examination of personality variables (Friede and Ryan, 2005; Michel and Clark, 2009; Michel et al., 2011) and job outcomes (Ceschi et al., 2016) in that context. Personality has been treated as both a mediator and a moderator between antecedents and work-family conflict (Wayne et al., 2004; Ceschi et al., 2016). According to Stoeva et al. (2002), negative affect mediates the relationship between stress and work-family conflict, and negativity moderates the relationship such that the relationship between stress and work-family conflict is stronger for individuals with high negative affect than it is for those with low negative affect.

Kant et al. (2013) suggested that leaders’ negative behaviors are linked to subordinates’ anxiety. Anxious subordinates perceive others negatively and are likely to increase their expressions of criticism and disapproval (Forgas and Vargas, 1998; Story and Repetti, 2006). Despotic leadership refers to aggressive behavior toward subordinates and to the exploitation that creates fear and stress among subordinates regarding their position in the organization (De Hoogh and Den Hartog, 2008). Those who face such issues at work carry the resulting emotional instability back home, where they often retreat from the family (Schulz and Martire, 2004; Story and Repetti, 2006) and are unavailable to help or support their families.

We argue that anxiety is high among subordinates who perceive their leaders’ behavior as exploitative and unfair (Kant et al., 2013) and that this dyadic relationship eventually affects their personal domains. Therefore, subordinates who have a high degree of anxiety are more likely to experience work-family conflict and diminished life satisfaction than are those who have less anxiety.

H5: Subordinates anxiety moderates the relationship between their perception of despotic leadership and (a) their work-family conflict and (b) their life satisfaction.

### Proposed Research Model

**FIGURE 1:**
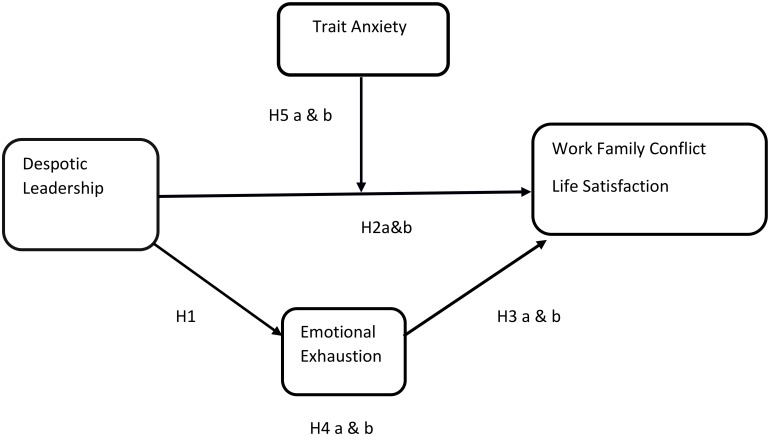
Proposed Theoretical Model.

**Figure 1** presents a model of our hypothesized relationships.

## Materials and Methods

### Sample and Data Collection Procedure

To reduce common method variance, the three-wave data was collected (Podsakoff et al., 2003). The respondents were salespeople working in book-publishing houses. The publishing houses were approached through personal contacts to identify respondents, and surveys were distributed by the researchers to the respondents in person who agreed to participate in the survey voluntarily. Prior to their participation in the surveys, the participants completed consent forms that explained the purpose of the study and assured complete confidentiality. It was communicated that all the responses would be accessible to the researchers only, no individual level information would be made public and only aggregate information would be shared. These precautions helped us to deal with social desirability and made the respondents feel confident. The late respondents and non-respondents were contacted in follow-up to increase the response rate. The study was approved by the Riphah International University Ethical Research Committee.

The data collection was completed by means of three pen-and-pencil surveys fielded on site, one of which measured despotic leadership and anxiety, the second of which measured emotional exhaustion, and the third measured life satisfaction and work-family conflict (time-, strain-, and behavior-based conflict). After completing the surveys, participants placed them in sealed envelopes and returned them to the contact person. The researchers collected the filled responses from the contact person. No monetary reward or other incentive was offered to participants, and participation was voluntary.

Questionnaires were initially distributed to 400 salespersons, and 327 completed surveys were returned. The second survey was offered only to the 327 who returned the first survey, and 255 completed surveys were returned. These 255 received their third survey, and 245 were returned. The surveys were fielded 3–4 weeks apart. The final sample size after discarding incomplete questionnaires and matching three-time data was 224, for a final response rate of those who completed all three surveys of 56 percent. As schools and most organizations in Pakistan use English, all survey questions were in English. All respondents were males, and all had reached at least the graduate level to ensure their solid understanding of English language.

### Measures

All measures were adopted from extant studies that had tested them in a variety of cultures, countries, and work settings. The use of established standardized scales to measure the study’s variables reduces the likelihood of instrumentation errors (Luthans and Youssef, 2007). To establish convergent and discriminant validity, confirmatory factor analyses were conducted for all variables.

#### Despotic Leadership

We used a six-item scale developed by De Hoogh and Den Hartog (2008) to measure despotic leadership. The items included “My supervisor is punitive and has no pity or compassion,” My supervisor is in charge and does not tolerate disagreement or questions,” and “My supervisor gives orders.” Responses were given on a 5-point Likert scale, anchored at 1 for strongly disagree and 5 for strongly agree. The Cronbach’s alpha for this scale was 0.80. Convergent validity was also established because all items loaded in a range of 0.45 to 0.74 with average variance extracted (AVE) = 0.50.

#### Emotional Exhaustion

To assess employees’ emotional exhaustion, we used a nine- item scale developed by Pines and Aronson (1988). Responses were rated on a 5-point scale, where 1 = never and 5 = very often. Examples of items are “I feel emotionally exhausted” and “I feel that I can’t take it anymore.” The Cronbach’s alpha for this scale was 0.86. Convergent validity was also established because all items loaded in a range of 0.48 to 0.78 with AVE = 0.52.

#### Job-Related Anxiety

Following Wagner et al. (2014), we used a four-item measure of anxiety drawn from (Mackinnon et al., 1999). Respondents indicated the extent to which they generally feel facts of anxiety (e.g., “nervous,” “distressed”) in their jobs using a 5-item Likert scale, where 1 = very slightly or not at all to 5 = very much. The Cronbach’s alpha for this scale was 0.76. Convergent validity was also established because all items loaded in a range of 0.63 to 0.83 with AVE = 0.61.

#### Work-Family Conflict

Work-family conflict was measured using the nine-item scale developed by Carlson and Kacmar (2000). We compared the three-factor model of work family conflict (time-based, strain-based, and behavior-based) with one overall-factor model. The overall-factor model produced better results than the three-factor model did, and the three dimensions were highly correlated, so we used the overall-, one-factor model. Responses were measured on 5-point Likert scale, anchored at 1 for strongly disagree and 5 for strongly agree. The Cronbach’s alpha for this scale was 0.86. Convergent validity was also established because all items loaded in a range of 0.43 to 0.84 with AVE = 0.54.

#### Life Satisfaction

We used a 5-item scale from (Diener et al., 1985) to measure life satisfaction. The five items included “In most ways, my life is close to my ideal” and “I am satisfied with my life.” One item was dropped because of low factor loading. The Cronbach’s alpha for the remaining four items was 0.75. Convergent validity was also established because all items loaded in a range of 0.51 to 0.80 with AVE = 0.56.

## Results

Structure equation modeling (SEM) (Bollen, 1989) using AMOS 16 was employed to test the hypotheses and followed the two-step analytical strategy suggested by Anderson and Gerbing (1988). The first step involved a confirmatory factor analysis to develop an acceptable measurement model that defined the observed variables in terms of “true” latent variables (endogenous or exogenous) and a measurement-error term. At this stage, each latent variable was allowed to correlate freely with every other latent variable. In step two, we modified the measurement model to represent the hypothesized theoretical framework. This strategy provided an analytical method with which to identify a best-fit measurement model and an estimation of the proposed hypotheses. To test our mediation hypotheses, we also used the bootstrap technique using the confidence-interval method. Bootstrapping is a resampling method that involves creating a sampling distribution to estimate standard errors and to create the confidence intervals. Considered important for mediation analysis, bootstrapping is used to confirm the mediation effect because of its accuracy in computing confidence intervals for the mediation effect when the mediation effect is non-zero. It can be applied when the assumptions of large sample size and multivariate normality may not hold (Cheung and Lau, 2008).

**Table 1** shows the descriptive statistics, bi-variate correlations, reliability estimates, and AVEs. We performed a series of confirmatory factor analyses to establish the discriminant validity of the variables, tapped through the same source and time. In light of (Anderson and Gerbing, 1988, 1992) suggestions, we compared a two-factor unconstrained model with a single-factor constrained model for every possible pairing of variables from the same source. The results of these confirmatory factor analyses are presented in **Table 2**, which shows that, in every comparison, the unconstrained multiple-factor model provided a better fit than the single-factor model did. All fit indices are within the range of a good model fit (Kline, 2005; Steiger, 2007; Tabachnik and Fidell, 2007).

**Table 1:** Descriptive statistics, correlation and reliabilities.

	Mean	*SD*	AVE	1	2	3	4	5
(1) DL	2.71	1.01	0.50	(0.80)				
(2) EE	2.13	0.86	0.52	0.45^∗∗^	(0.86)			
(3) TA	2.68	0.98	0.61	0.37^∗∗^	0.31^∗∗^	(0.76)		
(4) WFC	2.68	0.89	0.54	0.43^∗∗^	0.54^∗∗^	0.29^∗∗^	(0.86)	
(5) LS	4.27	1.36	0.56	-0.20^∗∗^	-0.09^∗^	-0.18^∗∗^	0.07	(0.75)
(6) Age	30	4.8	–	0.13^∗^	0.006	0.09	0.07	-0.15^∗^

*N = 224. ^∗^Correlation is significant at the 0.05 level (two-tailed). ^∗∗^Correlation is significant at the 0.01 level (two-tailed). DL, Despotic Leadership; EE, Emotional Exhaustion; TA, Trait Anxiety; LS, Life Satisfaction; WFC, Work family conflict.*

**Table 2:** Model Fit Indices for CFAs.

Model Test	χ2	df	χ2/df	CFI	NFI	GFI	TLI	RMR	RMSEA
***For T1***		
1 Factor (DL and TA combined)	213	35	6.08	0.72	0.69	0.80	0.64	0.18	0.15
**2 factor (DL, TA)**	**62**	**34**	**1.83**	**0.95**	**0.91**	**0.94**	**0.94**	**0.094**	**0.06**
**1 factor WFC(All dimensions)**	**26.05**	**11**	**2.3**	**0.98**	**0.96**	**0.97**	**0.93**	**0.04**	**0.07**
3 Factor WFC(WFCTM, WFCSTR, WFCBHR)	91.08	24	3.79	0.91	0.89	0.91	0.87	0.103	0.112
***ALL DVs T2***		
1 factor (WFC, LS combined)	303	62	4.88	0.79	0.75	0.85	0.69	0.27	0.13
**2 factor (WFC, LS)**	**142**	**52**	**2.73**	**0.91**	**0.88**	**0.91**	**0.87**	**0.168**	**0.08**
1 factor(DL, EE, WFC, LS)	867	328	2.6	0.80	0.72	0.79	0.77	0.16	0.08
**4 factor(DL, EE, WFC, LS)**	**477**	**295**	**1.6**	**0.93**	**0.85**	**0.87**	**0.91**	**0.15**	**0.05**
1 factor EE, TA Combined	294	61	4.8	0.78	0.74	0.82	0.72	0.16	0.13
**2 factor (EE, TA)**	**140**	**60**	**2.3**	**0.92**	**0.87**	**0.91**	**0.90**	**0.10**	**0.07**
**5 factor (DL, EE, TA, WFC, and LS)**	**639**	**389**	**1.64**	**0.93**	**0.90**	**0.87**	**0.91**	**0.15**	**0.05**
1 factor (DL, EE, TA, WFC, and LS Combined)	648	410	4.02	0.62	0.63	0.56	0.54	0.23	0.11

*N = 224. T1, time 1; T2, time 2. DL, Despotic Leadership; EE, Emotional Exhaustion; TA, Trait Anxiety; LS, Life Satisfaction; WFC, Work family conflict. Best model fits are given in bold.*

**FIGURE 2:**
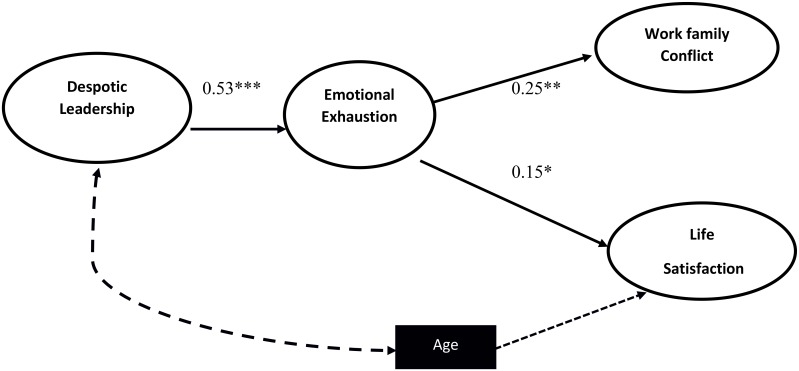
Hypothesized Mediation Model. *N* = 224. DL, Despotic Leadership; EE, Emotional Exhaustion; TA, Trait Anxiety; LS, Life Satisfaction. Age is controlled for Despotic Leadership and Life Satisfaction.

After getting adequate model fit results for the measurement models, we estimated the path models using SEM to test the hypotheses. **Table 3** shows the results of SEM analyses, and **Figure 2** presents the standardized path coefficients of the best fitting model. We tested three structural models to determine which provided the best fit to data. The first model included indirect paths between despotic leadership and two outcomes—work-family conflict and life satisfaction through emotional exhaustion. The second model included direct and indirect paths between despotic leadership and the same two outcomes through emotional exhaustion and revealed a direct path between despotic leadership and the two outcome variables. The results indicated that the indirect-path model between despotic leadership and the two outcomes through emotional exhaustion provided the best results for model fit indices (χ2 = 549.89, df = 324, χ2/df = 1.69, CFI = 0.92, NFI = 0.88, GFI = 0.90, TLI = 0.90, RMR = 0.19, and RMSEA = 0.05), as shown in **Table 3**.

**Table 3:** Comparison of alternative path models.

Model Test	χ2	df	χ2/df	CFI	NFI	GFI	TLI	RMR	RMSEA
1 Hypothesized Model: Indirect paths from DL to outcomes through EE)	549.89	324	1.69	0.92	0.88	0.87	0.90	0.19	0.05
2 Alternative Model 1: Indirect paths from DL to outcomes through EE and direct pat from DL to outcomes	597	325	1.83	0.90	0.81	0.85	0.88	0.20	0.06
3 Alternative Model 2: Direct Path from DL to outcomes	321.44	147	2.18	0.89	0.86	0.88	0.83	0.20	0.07

*N = 224. Age is controlled in all models. DL, Despotic Leadership; EE, Emotional Exhaustion; Outcomes, life satisfaction and work family conflict.*

The direct-path model provides the results for H1, which predicts that despotic leadership is positively related to work-family conflict and negatively related to life satisfaction. The results provide support for both work-family conflict (*β* = 0.55, *p* < 0.001) and life satisfaction (*β* = -0.27, *p* < 0.001). In support of H2, despotic leadership is positively related to emotional exhaustion (*β* = 0.53, *p* < 0.001). Hypotheses 3a and 3b, that emotional exhaustion has a positive relationship with work-family conflict (*β* = 0.25, *p* < 0.01) and a negative relationship with life satisfaction (*β* = -0.15, *p* < 0.05), are also supported. Hypotheses 4a and 4b predict a mediating role of emotional exhaustion between despotic leadership and both outcome variables. We applied bootstrapping using a bias-corrected confidence interval method to analyze the indirect effects. The results support the indirect effect of work-family conflict (*indirect effect* = 0.13, CI 95%, [0.04,0.27], *p* ≤ 0.01) but not life satisfaction (*indirect effect* = -0.08, CI95%, [-0.20,0.01], *p* > 0.05). All these direct and indirect effects are given in **Table 4**.

**Table 4:** Standardized direct path coefficients of the hypothesized model.

	Path	Estimate		SE	
H1 (a)	DL→WFC	0.55^∗∗∗^		0.08	
(b)	DL→LS	-0.27^∗∗∗^		0.08	
H2	DL→EE	0.53^∗∗∗^		0.05	
H3 (a)	EE→WFC	0.25^∗∗^		0.06	
(b)	EE→LS	-0.15^∗^		0.08	

	**Bootstrap Results for Indirect Effects**				
	**(Bias Corrected Confidence Interval Method)**				
	**Paths**	**Effect**	**SE**	**LL 99%CI**	**UL 99%CI**

H4 a	DL→EE→WFC	0.138	0.01	0.04	0.27
H4 b	DL→EE→LS	-0.08	0.03	-0.20	

*N = 224. Bootstrap sample size = 5,000. Age is controlled in all models. ^∗∗∗^p ≤ 0.001, ^∗∗^p ≤ 0.01, ^∗^p ≤ 0.05. T1, time 1; T2, time 2. DL, Despotic Leadership; EE, Emotional Exhaustion; LS, Life Satisfaction; WFC, Work family conflict; LL, lower limit; CI, confidence interval; UL, upper limit.*

**FIGURE 3:**
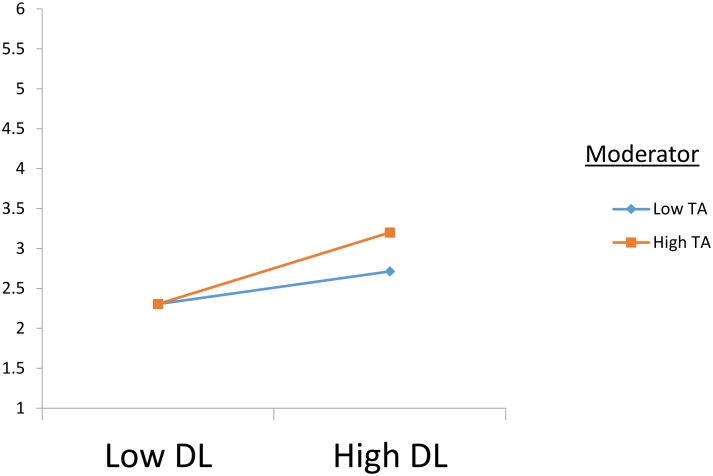
Interaction plot for WFC. DL, Despotic Leadership; TA, Trait Anxiety; WFC, Work Family Conflict. *N* = 224.

**FIGURE 4:**
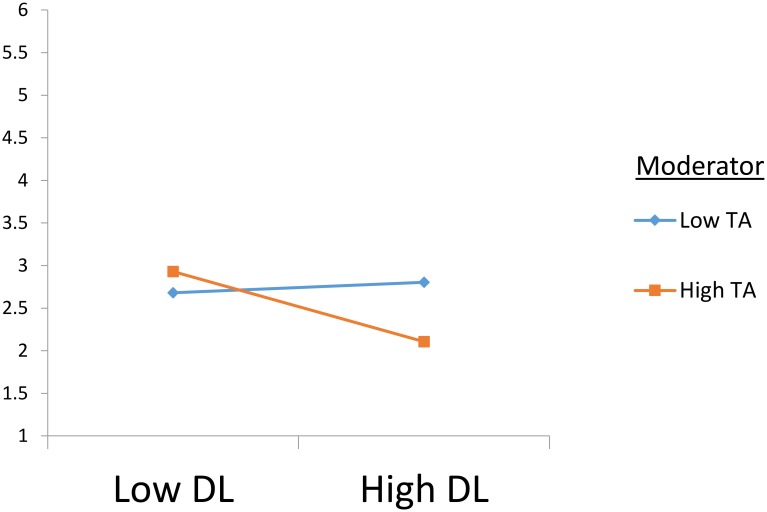
Interaction plot for life satisfaction. DL, Despotic Leadership; TA, Trait Anxiety; LS, Life Satisfaction. *N* = 224.

Hypotheses 5a and 5b propose a moderating role of anxiety between despotic leadership and work-family conflict and life satisfaction. Our moderation analysis employed Hayes’ 13 macro PROCESS, and mean-centering was done for the independent and moderation variables (Aiken and West, 1991). The results indicate support for both variables, work-family conflict (β = 0.09, *p* < 0.05, CI [0.01,0.19], Δ*R*^2^ = 0.015) and life satisfaction (β = -0.23, *p* < 0.001, CI [-0.38,-0.08], Δ*R*^2^ = 0.037). The slope test indicates that the change in beta is in the same direction as proposed, at ±1 standard deviation of the moderator’s mean value. The interaction plots shown in **Figures 3**, **4** show that the interaction for work-family conflict is stronger when anxiety is high (i.e., β = 0.42, *p* < 0.001) than when it is low (β = 0.23, *p* < 0.001). Similarly, the interaction for life satisfaction is negative when anxiety is high (β = -0.40, *p* < 0.001), whereas it becomes insignificant when anxiety is low (β = -0.05, *p* > 0.05) as given in **Table 5**. These results are in line with our proposed moderation hypotheses.

**Table 5:** Moderation analysis.

Moderation Analysis Results (Bootstrap 95%Confidence Interval)								
	Life Satisfaction				Work Family Conflict			
	β	*SE*	LLCI	ULCI	β	*SE*	LLCI	ULCI
Constant	5.77	0.55	4.69	6.86	2.63	0.33	1.96	3.30
TA	-0.112^∗^	0.09	-0.30	-0.07	0.122^∗^	0.05	0.01	0.23
DL	-0.175^∗∗^	0.09	-0.35	-0.01	0.32^∗∗∗^	0.05	0.21	0.43
TAxDL	-0.236^∗∗∗^	0.07	-0.38	-0.08	0.09^∗^	0.04	0.01	0.19
Δ*R*^2^ due to Interaction	0.037^∗∗∗^				0.015^∗^			

**Slope Test**								
Moderator: TA								
-0.98	-0.05	0.121	-0.185	0.295	0.231^∗∗∗^	0.07	0.08	0.37
0.00	-0.17^∗∗∗^	0.093	-0.359	0.007	0.326^∗∗∗^	0.05	0.21	0.43
+0.98	-0.40^∗∗∗^	0.117	-0.639	-0.174	0.421^∗^	0.07	0.27	0.56

*N = 224. Unstandardized regression coefficients are reported. DL, Despotic Leadership; TA, Trait Anxiety; Bootstrap sample size = 5,000. LL, lower limit; CI, confidence interval; UL, upper limit. ^∗^p ≤ 0.05, ^∗∗^p ≤ 0.01, ^∗∗∗^p ≤ 0.001.*

## Discussion

By integrating despotic leadership, anxiety, work-family conflict, and life satisfaction with the CORs theory, we find evidence of an indirect effect of despotic leadership on work-family conflict and life satisfaction via emotional exhaustion. Our findings indicate that emotional exhaustion partially mediates the relationship of despotic leadership with work-family conflict and life satisfaction. We also show that highly anxious employees are more prone respond negatively to despotic leadership, increasing work-family conflict and decreasing life satisfaction. These findings, which are in line with previous research (Story and Repetti, 2006; Carlson et al., 2011, 2012; Kant et al., 2013), suggest that despotic leaders have harmful effects on their subordinates’ home lives and that these effects intensify when subordinates are anxious. These findings are in line with previous research. Given these findings, this study contributes to the literature on dark side of leadership, employee wellbeing, and CORs theory. This study also revealed a significant correlation between age of the subordinates and perceived despotic leadership and life satisfaction. The demographic variables are supposed to be controlled in studies intended to measure employee attitudes and behaviors (Riordan et al., 2003). Therefore, we controlled age in the mediation model.

### Theoretical Implications

This study makes theoretical contributions to both the dark side of leadership literature and the work-family literature. We extend both the despotic leadership and work-family literatures by investigating the relationships between despotic leadership and subordinates’ work-family conflict and life satisfaction in Pakistan’s cultural setting, which is ranked high in collectivism, uncertainty avoidance, and power distance (Hofstede, 1983). Our research shows that the negative effects of despotic leadership extend beyond the work domain to affect subordinates’ non-work lives. We also provide insights into how despotic leadership relates to the family domain via emotional exhaustion. The interactions between despotic leaders and their subordinates leave subordinates emotionally exhausted and prone to work-family conflict and low life satisfaction. Finally, we identify a boundary condition drawn by anxiety on the relationship between despotic leadership and the family domain.

### Practical Implications

An important practical implication is that despotic leadership has detrimental effects on the home lives and life satisfaction of subordinates who work in the service sector. An organization that fails to identify leaders who have despotic tendencies and an overarching desire for power risks having emotionally exhausted and dissatisfied employees. The ideal is for organizations to avoid appointing such leaders in the first place, but steps can also be taken to reduce subordinates’ emotional exhaustion by giving them easy access to the human resources department, where they can give confidential feedback about the despotic supervisor. Confidentiality is essential as despotic leaders are likely to manipulate and harm subordinates who give such feedback. When feedback is provided about despotic leaders, grievances should be addressed by means of appropriate investigation. Checks and balances can help to prevent despotic leadership (Padilla et al., 2007). As booksellers’ work is incentive-based, reward and incentives like company recognition and job-promotion opportunities can help to increase subordinates’ self-esteem (Ceschi et al., 2017) and reduce emotional exhaustion. Interventions like psychological training can also reduce emotional exhaustion and work disengagement (Costantini et al., 2017).

Another practical implication is that the harmful effects of despotic leadership on subordinates can be attenuated if HR fosters positive organizational climate for reducing despotic leadership and provides support to the subordinates who suffered. Engaging employees in such recovery activities as relaxation, personal control, psychological detachment, and exercise can help them reduce the anxiety that leads to work-life conflict and life dissatisfaction (Erfurt et al., 1992; Sonnentag et al., 2008, 2010).

### Strengths, Limitations and Suggestions for Future Research

A major limitation to the present study is that all participants were male, and all were drawn from two publishing houses. Future research should examine both genders and additional occupations to determine the extent to which the findings can be generalized.

Another limitation is that all of the data was collected through self-reports. Studies that rely on self-reports can suffer from common method variance (Podsakoff et al., 2003), However, the study’s research design minimizes such concerns, as the data for the independent variable and the moderator, that for the mediator, and that for work-family conflict and life satisfaction were collected at different times that helped to avoid common method variance (Podsakoff et al., 2012). A study on addressing the issues of common method variance by Johnson et al. (2011) found that 3-week gap between predictors and criterion variables decreased correlations between constructs by 43 percent. Moreover, nature of all variables require self-reports of the subordinates like rating the perceived despotic leadership. The time-lagged design also addresses reverse causality between variables in mediation models. Moreover, the correlation size from low to moderate reveals that there is no issue of common method variance among all study variables. To improve the results’ accuracy, all of the data were collected from the same employees and matched time lagged responses. As participants responded to the survey voluntarily and at their convenience, we have considered the possibility of a self-selection bias. However, as the response rate across three waves of data collection was comparatively high, we argue that a strong influence of a self-selection bias on the subsequent results is unlikely.

The strength of the study is that it was conducted in Pakistan, which provides an ideal context in which to examine the negative aspects of despotic leadership (Naseer et al., 2016). Subordinates in high power-distant and collectivistic cultures are expected to do what they are told to do by their supervisors and accept power inequalities. However, despotic leaders goes beyond controlling and self-serving behavior and are engaged in exploitative and unethical acts which can drain subordinates resources and heighten emotional exhaustion, reducing life satisfaction and increasing work-family conflict. Subordinates in certainty-avoidant cultures like that of Pakistan also prefer to stay in their jobs—even those they dislike—so they seldom do anything about the inter-role conflict that results in work-family conflict and life dissatisfaction and thus suffer in their home lives, especially if they have high levels of anxiety.

As the data for the current study was collected at three points in time, future researchers may consider measuring the daily effects of despotic leadership. Future research could also examine other factors that may influence the day-to-day variance in the effects of despotic leadership, such as lack of sleep. There is also room to clarify this interface further by examining additional moderators of such relationships, such as emotional intelligence, organizational justice, faith and social support.

## Conclusion

This study extends research on the dark side of leadership by showing that despotic leadership has negative effects on work-family conflict and life satisfaction. Despotic leadership is related to work-family conflict via emotional exhaustion, and the interaction of anxiety with despotic leadership has negative effects on work-family conflict and life satisfaction. Thus, despotic leadership socially undermines subordinates. This study should stimulate research on how to control and attenuate the harmful impacts of despotic leadership on employees in service organizations in order to enhance their wellbeing.

## Ethics Statement

Research Ethics Committee at Riphah International University has reviewed the aforementioned research proposal and considers the procedures, as described by the applicant, to conform to the University’s ethical standards and RIU guidelines. The committee has granted clearance from 03/02/2017 to 01/02/2018.

## Author Contributions

All authors listed have made a substantial, direct, and intellectual contribution to the work, and approved it for publication.

## Conflict of Interest Statement

The authors declare that the research was conducted in the absence of any commercial or financial relationships that could be construed as a potential conflict of interest.

## References

[B1] Aasland M. S. Skogstad A. Notelaers G. Nielsen M. B. Einarsen S. (2010). The prevalence of destructive leadership behaviour. *Br. J. Manag.* 21 438–452.

[B2] Aiken L. S. West S. G. (1991). *Multiple Regression: Testing and Interpreting Interactions.* Newbury Park, CA: Sage.

[B3] Anderson J. C. Gerbing D. W. (1988). Structural equation modeling in practice: a review and recommended two-step approach. *Psychol. Bull.* 103:411 10.1037/0033-2909.103.3.411

[B4] Anderson J. C. Gerbing D. W. (1992). Assumptions and comparative strengths of the two-step approach: comment on Fornell and Yi. *Sociol. Methods Res.* 20 321–333. 10.1177/0049124192020003002

[B5] Aquino K. Thau S. (2009). Workplace victimization: aggression from the target’s perspective. *Annu. Rev. Psychol.* 60 717–741. 10.1146/annurev.psych.60.110707.163703 19035831

[B6] Aronson E. (2001). Integrating leadership styles and ethical perspectives. *Can. J. Adm. Sci.* 18 244–256. 10.1111/j.1936-4490.2001.tb00260.x

[B7] Aryee S. Chen Z. X. Sun L.-Y. Debrah Y. A. (2007). Antecedents and outcomes of abusive supervision: test of a trickle-down model. *J. Appl. Psychol.* 92 191–201. 10.1037/0021-9010.92.1.191 17227160

[B8] Aryee S. Sun L. Y. Chen Z. X. G. Debrah Y. A. (2008). Abusive supervision and contextual performance: the mediating role of emotional exhaustion and the moderating role of work unit structure. *Manage. Organ. Rev.* 4 393–411. 10.1111/j.1740-8784.2008.00118.x

[B9] Ashforth B. (1994). Petty tyranny in organizations. *Hum. Relat.* 47 755–778. 10.1177/001872679404700701

[B10] Ashforth B. E. (1997). Petty tyranny in organizations: a preliminary examination of antecedents and consequences. *Can. J. Adm. Sci.* 14 126–140. 10.1111/j.1936-4490.1997.tb00124.x

[B11] Ashforth B. E. Lee R. T. (1997). Burnout as a process: commentary on Cordes, Dougherty and Blum. *J. Organ. Behav.* 18 703–708. 10.1002/(SICI)1099-1379(199711)18:6<703::AID-JOB847>3.0.CO;2-1

[B12] Bass B. M. (1990). From transactional to transformational leadership: learning to share the vision. *Organ. Dyn.* 18 19–31. 10.1016/0090-2616(90)90061-S

[B13] Boekhorst J. A. Singh P. Burke R. (2017). Work intensity, emotional exhaustion and life satisfaction: the moderating role of psychological detachment. *Pers. Rev.* 46 891–907. 10.1108/PR-05-2015-0130

[B14] Bollen K. A. (1989). A new incremental fit index for general structural equation models. *Sociol. Methods Res.* 17 303–316. 10.1177/0049124189017003004

[B15] Carlson D. Ferguson M. Hunter E. Whitten D. (2012). Abusive supervision and work–family conflict: the path through emotional labor and burnout. *Leadersh. Q.* 23 849–859. 10.1016/j.leaqua.2012.05.003

[B16] Carlson D. S. Ferguson M. Perrewé P. L. Whitten D. (2011). The fallout from abusive supervision: an examination of subordinates and their partners. *Pers. Psychol.* 64 937–961. 10.1111/j.1744-6570.2011.01232.x

[B17] Carlson D. S. Kacmar K. M. (2000). Work–family conflict in the organization: do life role values make a difference? *J. Manag.* 26 1031–1054. 10.1177/014920630002600502

[B18] Ceschi A. Costantini A. Dickert S. Sartori R. (2017). The impact of occupational rewards on risk taking among managers. *J. Pers. Psychol.* 16 104–111. 10.1027/1866-5888/a000184

[B19] Ceschi A. Sartori R. Dickert S. Costantini A. (2016). Grit or honesty-humility? New insights into the moderating role of personality between the health impairment process and counterproductive work behavior. *Front. Psychol.* 7:1799. 10.3389/fpsyg.2016.01799 28018250 PMC5147463

[B20] Chen J.-C. Lu K.-W. Tsai M.-L. Hsu S.-C. Kuo C.-L. Yang J.-S. (2009). Gypenosides induced G0/G1 arrest via CHk2 and apoptosis through endoplasmic reticulum stress and mitochondria-dependent pathways in human tongue cancer SCC-4 cells. *Oral Oncol.* 45 273–283. 10.1016/j.oraloncology.2008.05.012 18674953

[B21] Cheung G. W. Lau R. S. (2008). Testing mediation and suppression effects of latent variables: bootstrapping with structural equation models. *Organ. Res. Methods* 11 296–325. 10.1177/1094428107300343

[B22] Clark S. C. (2000). Work/family border theory: a new theory of work/family balance. *Hum. Relat.* 53 747–770. 10.1177/0018726700536001

[B23] Collins M. D. Jackson C. J. (2015). A process model of self-regulation and leadership: how attentional resource capacity and negative emotions influence constructive and destructive leadership. *Leadersh. Q.* 26 386–401. 10.1016/j.leaqua.2015.02.005

[B24] Costantini A. De Paola F. Ceschi A. Sartori R. Meneghini A. M. Di Fabio A. (2017). Work engagement and psychological capital in the Italian public administration: a new resource-based intervention programme. *SA J. Ind. Psychol.* 43 1–11. 10.4102/sajip.v43i0.1413

[B25] De Hoogh A. H. Den Hartog D. N. (2008). Ethical and despotic leadership, relationships with leader’s social responsibility, top management team effectiveness and subordinates’ optimism: a multi-method study. *Leadersh. Q.* 19 297–311. 10.1016/j.leaqua.2008.03.002

[B26] Demsky C. A. Ellis A. M. Fritz C. (2014). Shrugging it off: does psychological detachment from work mediate the relationship between workplace aggression and work-family conflict? *J. Occup. Health Psychol.* 19 195–205. 10.1037/a0035448 24635738

[B27] Den Hartog D. N. De Hoogh A. H. (2009). Empowering behaviour and leader fairness and integrity: studying perceptions of ethical leader behaviour from a levels-of-analysis perspective. *Eur. J. Work Organ. Psychol.* 18 199–230. 10.1080/13594320802362688

[B28] Diener E. Emmons R. A. Larsen R. J. Griffin S. (1985). The satisfaction with life scale. *J. Pers. Assess.* 49 71–75. 10.1207/s15327752jpa4901_13 16367493

[B29] Duffy M. K. Ganster D. C. Pagon M. (2002). Social undermining in the workplace. *Acad. Manage. J.* 45 331–351. 10.2307/3069350

[B30] Dzuka J. Dalbert C. (2007). Student violence against teachers: teachers’ well-being and the belief in a just world. *Eur. Psychol.* 12 253–260. 10.1027/1016-9040.12.4.253

[B31] Eby L. T. Maher C. P. Butts M. M. (2010). The intersection of work and family life: the role of affect. *Annu. Rev. Psychol.* 61 599–622. 10.1146/annurev.psych.093008.100422 19572785

[B32] Erdogan B. Bauer T. N. Truxillo D. M. Mansfield L. R. (2012). Whistle while you work: a review of the life satisfaction literature. *J. Manag.* 38 1038–1083. 10.1177/0149206311429379

[B33] Erfurt J. C. Foote A. Heirich M. (1992). The cost-effectiveness of worksite wellness programs for hypertension control, weight loss, smoking cessation, and exercise. *Pers. Psychol.* 45 5–27. 10.1111/j.1744-6570.1992.tb00842.x 1744745

[B34] Ernst Kossek E. Ozeki C. (1998). Work–family conflict, policies, and the job–life satisfaction relationship: a review and directions for organizational behavior–human resources research. *J. Appl. Psychol.* 83 139–149. 10.1037/0021-9010.83.2.139

[B35] Fontaine P. Ross S. E. Zink T. Schilling L. M. (2010). Systematic review of health information exchange in primary care practices. *J. Am. Board Fam. Med.* 23 655–670. 10.3122/jabfm.2010.05.090192 20823361

[B36] Forgas J. P. Vargas P. (1998). Affect and behavior inhibition: the mediating role of cognitive processing strategies. *Psychol. Inq.* 9 205–210. 10.1207/s15327965pli0903_2 23370083

[B37] Friede A. Ryan A. M. (2005). *The Importance of the Individual: How Self-Evaluations Influence the Work-Family Interface.* Mahwah, NJ: Lawrence Erlbaum Associates Publishers.

[B38] Frone M. R. (2003). Predictors of overall and on-the-job substance use among young workers. *J. Occup. Health Psychol.* 8 39–54. 10.1037/1076-8998.8.1.39 12553528

[B39] Gali Cinamon R. Rich Y. (2010). Work family relations: antecedents and outcomes. *J. Career Assess.* 18 59–70. 10.1177/1069072709340661

[B40] Grandey A. A. Cropanzano R. (1999). The conservation of resources model applied to work–family conflict and strain. *J. Vocat. Behav.* 54 350–370. 10.1006/jvbe.1998.1666

[B41] Grandey A. A. Dickter D. N. Sin H. P. (2004). The customer is not always right: customer aggression and emotion regulation of service employees. *J. Organ. Behav.* 25 397–418. 10.1002/job.252

[B42] Greenhaus J. H. Beutell N. J. (1985). Sources of conflict between work and family roles. *Acad. Manage. Rev.* 10 76–88.

[B43] Greenhaus J. H. Collins K. M. Shaw J. D. (2003). The relation between work–family balance and quality of life. *J. Vocat. Behav.* 63 510–531. 10.1016/S0001-8791(02)00042-8

[B44] Griffin R. W. Lopez Y. P. (2005). “Bad behavior” in organizations: a review and typology for future research. *J. Manag.* 31 988–1005. 10.1177/0149206305279942

[B45] Harvey P. Stoner J. Hochwarter W. Kacmar C. (2007). Coping with abusive supervision: the neutralizing effects of ingratiation and positive affect on negative employee outcomes. *Leadersh. Q.* 18 264–280. 10.1016/j.leaqua.2007.03.008

[B46] Hershcovis M. S. Rafferty A. E. (2012). Predicting abusive supervision. *Contemp. Occup. Health Psychol. Glob. Perspect. Res. Pract.* 2 92–108. 10.1002/9781119942849.ch6

[B47] Hobfoll S. E. (1989). Conservation of resources: a new attempt at conceptualizing stress. *Am. Psychol.* 44 513–524. 10.1037/0003-066X.44.3.513 2648906

[B48] Hofstede G. (1983). The cultural relativity of organizational practices and theories. *J. Int. Bus. Stud.* 14 75–89. 10.1057/palgrave.jibs.8490867

[B49] Hofstede G. (2010). *National Cultural Dimensions.* New York City, NY: McGraw-Hill Education.

[B50] Hoobler J. M. Brass D. J. (2006). Abusive supervision and family undermining as displaced aggression. *J. Appl. Psychol.* 91 1125–1533. 10.1037/0021-9010.91.5.1125 16953773

[B51] Hoobler J. M. Hu J. (2013). A model of injustice, abusive supervision, and negative affect. *Leadersh. Q.* 24 256–269. 10.1016/j.leaqua.2012.11.005

[B52] Howell J. M. Avolio B. J. (1992). The ethics of charismatic leadership: submission or liberation? *Executive* 6 43–54. 10.5465/AME.1992.4274395

[B53] Ilies R. Wilson K. S. Wagner D. T. (2009). The spillover of daily job satisfaction onto employees’ family lives: the facilitating role of work-family integration. *Acad. Manage. J.* 52 87–102. 10.5465/AMJ.2009.36461938

[B54] Johnson H.-A. M. Spector P. E. (2007). Service with a smile: do emotional intelligence, gender, and autonomy moderate the emotional labor process? *J. Occup. Health Psychol.* 12 319–333. 10.1037/1076-8998.12.4.319 17953492

[B55] Johnson R. E. Rosen C. C. Djurdjevic E. (2011). Assessing the impact of common method variance on higher order multidimensional constructs. *J. Appl. Psychol.* 96 744–761. 10.1037/a0021504 21142343

[B56] Kalliath P. Hughes M. Newcombe P. (2012). When work and family are in conflict: impact on psychological strain experienced by social workers in Australia. *Aust. Soc. Work* 65 355–371. 10.1080/0312407X.2011.625035

[B57] Kant L. Skogstad A. Torsheim T. Einarsen S. (2013). Beware the angry leader: trait anger and trait anxiety as predictors of petty tyranny. *Leadersh. Q.* 24 106–124. 10.1016/j.leaqua.2012.08.005

[B58] Karatepe O. M. Baddar L. (2006). An empirical study of the selected consequences of frontline employees’ work–family conflict and family–work conflict. *Tour. Manag.* 27 1017–1028. 10.1016/j.tourman.2005.10.024

[B59] Kline T. (2005). *Psychological Testing: A Practical Approach to Design and Evaluation.* Thousand Oaks, CA: Sage.

[B60] Lambert E. G. Altheimer I. Hogan N. L. (2010). Exploring the relationship between social support and job burnout among correctional staff. *Crim. Justice Behav.* 37 1217–1236. 10.1177/0093854810379552

[B61] Luthans F. Peterson S. J. Ibrayeva E. (1998). The potential for the “dark side” of leadership in post communist countries. *J. World Bus.* 33 185–201. 10.1016/S1090-9516(98)90005-0

[B62] Luthans F. Youssef C. M. (2007). Emerging positive organizational behavior. *J. Manag.* 33 321–349. 10.1177/0149206307300814

[B63] Mackinnon A. Jorm A. F. Christensen H. Korten A. E. Jacomb P. A. Rodgers B. (1999). A short form of the positive and negative affect schedule: evaluation of factorial validity and invariance across demographic variables in a community sample. *Pers. Individ. Dif.* 27 405–416. 10.1016/S0191-8869(98)00251-7

[B64] Maslach C. Schaufeli W. B. Leiter M. P. (2001). Job burnout. *Annu. Rev. Psychol.* 52 397–422. 10.1146/annurev.psych.52.1.397 11148311

[B65] Michel J. S. Clark M. A. (2009). Has it been affect all along? A test of work-to-family and family-to-work models of conflict, enrichment, and satisfaction. *Pers. Individ. Dif.* 47 163–168. 10.1016/j.paid.2009.02.015

[B66] Michel J. S. Kotrba L. M. Mitchelson J. K. Clark M. A. Baltes B. B. (2011). Antecedents of work–family conflict: a meta-analytic review. *J. Organ. Behav.* 32 689–725. 10.1002/job.695

[B67] Moore P. M. Huebner E. S. Hills K. J. (2012). Electronic bullying and victimization and life satisfaction in middle school students. *Soc. Indic. Res.* 107 429–447. 10.1007/s11205-011-9856-z 27339866

[B68] Naseer S. Raja U. Syed F. Donia M. B. Darr W. (2016). Perils of being close to a bad leader in a bad environment: exploring the combined effects of despotic leadership, leader member exchange, and perceived organizational politics on behaviors. *Leadersh. Q.* 27 14–33. 10.1016/j.leaqua.2015.09.005

[B69] Olweus D. (1978). *Aggression in the Schools: Bullies and Whipping Boys.* Oxford: Hemisphere.

[B70] Padilla A. Hogan R. Kaiser R. B. (2007). The toxic triangle: destructive leaders, susceptible followers, and conducive environments. *Leadersh. Q.* 18 176–194. 10.1016/j.leaqua.2007.03.001

[B71] Pavot W. Diener E. (1993). Review of the satisfaction with life scale. *Psychol. Assess.* 5 164–172. 10.1037/1040-3590.5.2.164

[B72] Pines A. Aronson E. (1988). *Career Burnout: Causes and Cures.* New York City, NY: Free Press.

[B73] Podsakoff P. M. MacKenzie S. B. Podsakoff N. P. (2012). Sources of method bias in social science research and recommendations on how to control it. *Annu. Rev. Psychol.* 63 539–569. 10.1146/annurev-psych-120710-100452 21838546

[B74] Podsakoff P. M. MacKenzie S. B. Podsakoff N. P. Lee J. Y. (2003). The mismeasure of man (agement) and its implications for leadership research. *Leadersh. Q.* 14 615–656. 10.1016/j.leaqua.2003.08.002

[B75] Rain J. S. Lane I. M. Steiner D. D. (1991). A current look at the job satisfaction/life satisfaction relationship: review and future considerations. *Hum. Relat.* 44 287–307. 10.1177/001872679104400305

[B76] Richman J. A. Flaherty J. A. Rospenda M. (1992). Mental health consequences. *JAMA* 267 692–694. 10.1001/jama.1992.03480050096032 1731137

[B77] Riordan C. M. Griffith R. W. Weatherly E. W. (2003). Age and work-related outcomes: the moderating effects of status characteristics. *J. Appl. Soc. Psychol.* 33 37–57. 10.1111/j.1559-1816.2003.tb02072.x

[B78] Schilling J. (2009). From ineffectiveness to destruction: a qualitative study on the meaning of negative leadership. *Leadership* 5 102–128. 10.1177/1742715008098312

[B79] Schulz R. Martire L. M. (2004). Family caregiving of persons with dementia: prevalence, health effects, and support strategies. *Am. J. Geriatr. Psychiatry* 12 240–249. 10.1097/00019442-200405000-00002 15126224

[B80] Schyns B. Hansbrough T. (2010). *When Leadership goes Wrong: Destructive Leadership, Mistakes, and Ethical Failures.* Nerul: IAP.

[B81] Schyns B. Schilling J. (2013). How bad are the effects of bad leaders? A meta-analysis of destructive leadership and its outcomes. *Leadersh. Q.* 24 138–158. 10.1016/j.leaqua.2012.09.001

[B82] Sonnentag S. Kuttler I. Fritz C. (2010). Job stressors, emotional exhaustion, and need for recovery: a multi-source study on the benefits of psychological detachment. *J. Vocat. Behav.* 76 355–365. 10.1016/j.jvb.2009.06.005

[B83] Sonnentag S. Mojza E. J. Binnewies C. Scholl A. (2008). Being engaged at work and detached at home: a week-level study on work engagement, psychological detachment, and affect. *Work Stress* 22 257–276. 10.1080/02678370802379440

[B84] Spielberger C. D. Sydeman S. J. (1994). “State-trait anxiety inventory and state-trait anger expression inventory,” in *The Use of Psychological Testing for Treatment Planning and Outcome Assessment*, ed. Maruish M. E. (Hillsdale, NJ: Erlbaum Associates), 292–321.

[B85] Steiger J. H. (2007). Understanding the limitations of global fit assessment in structural equation modeling. *Pers. Individ. Dif.* 42 893–898. 10.1016/j.paid.2006.09.017

[B86] Stoeva A. Z. Chiu R. K. Greenhaus J. H. (2002). Negative affectivity, role stress, and work–family conflict. *J. Vocat. Behav.* 60 1–16. 10.1006/jvbe.2001.1812

[B87] Story L. B. Repetti R. (2006). Daily occupational stressors and marital behavior. *J. Fam. Psychol.* 20 690–700. 10.1037/0893-3200.20.4.690 17176205

[B88] Tabachnik B. Fidell S. (2007). *Discriminant Analysis: Using Multivariate Statistics*, Vol. 201 Boston, MA: Pearson Education Inc, 377–438

[B89] Tepper B. J. (2000). Consequences of abusive supervision. *Acad. Manage. J.* 43 178–190. 10.2307/1556375

[B90] Tepper B. J. (2007). Abusive supervision in work organizations: review, synthesis, and research agenda. *J. Manag.* 33 261–289. 10.1177/0149206307300812

[B91] Tepper B. J. Duffy M. K. Henle C. A. Lambert L. S. (2006). Procedural injustice, victim precipitation, and abusive supervision. *Pers. Psychol.* 59 101–123. 10.1111/j.1744-6570.2006.00725.x

[B92] Tepper B. J. Duffy M. K. Hoobler J. Ensley M. D. (2004). Moderators of the relationships between coworkers’ organizational citizenship behavior and fellow employees’ attitudes. *J. Appl. Psychol.* 89 455–465. 10.1037/0021-9010.89.3.455 15161405

[B93] Van Steenbergen E. F. Ellemers N. Mooijaart A. (2009). “How family supportive work environments and work supportive home environments can reduce work-family conflict and enhance facilitation,” in *Handbook of Families & Work: Interdisciplinary Perspectives*, eds Crane Russell D. Hill J. E. (Lanham, MD: University Press of America), 79–104.

[B94] Wagner D. T. Barnes C. M. Scott B. A. (2014). Driving it home: how workplace emotional labor harms employee home life. *Pers. Psychol.* 67 487–516. 10.1111/peps.12044

[B95] Wayne J. H. Musisca N. Fleeson W. (2004). Considering the role of personality in the work–family experience: relationships of the big five to work–family conflict and facilitation. *J. Vocat. Behav.* 64 108–130. 10.1016/S0001-8791(03)00035-6

[B96] Westman M. (2001). Stress and strain crossover. *Hum. Relat.* 54 717–751. 10.1177/0018726701546002

[B97] Westman M. Etzion D. Gortler E. (2004). The work-family interface and burnout. *Int. J. Stress Manag.* 11 413–428. 10.1037/1072-5245.11.4.413

[B98] Witt L. Carlson D. S. (2006). The work-family interface and job performance: moderating effects of conscientiousness and perceived organizational support. *J. Occup. Health Psychol.* 11 343–357. 10.1037/1076-8998.11.4.343 17059298

[B99] Wu T.-Y. Hu C. (2009). Abusive supervision and employee emotional exhaustion: dispositional antecedents and boundaries. *Group Organ. Manag.* 34 143–169. 10.1177/1059601108331217

